# ANALYSIS OF DAYTIME SLEEPINIESS IN ADOLESCENTS BY THE PEDIATRIC DAYTIME SLEEPINESS SCALE: A SYSTEMATIC REVIEW

**DOI:** 10.1590/1984-0462/;2017;35;3;00015

**Published:** 2017-07-31

**Authors:** Carolina Meyer, Geraldo Jose Ferrari, Diego Grasel Barbosa, Rubian Diego Andrade, Andreia Pelegrini, Érico Pereira Gomes Felden

**Affiliations:** a Universidade do Estado de Santa Catarina, Florianópolis, SC, Brasil.,

**Keywords:** Disorders of excessive somnolence, Evaluation, Sleep, Adolescent health, Adolescent

## Abstract

**Objective:**

To systematically review the use of the Pediatric Daytime Sleepiness Scale (PDSS) in the analysis of daytime sleepiness in children and adolescents.

**Data source:**

The electronic databases PubMed and SciELO were consulted between 2003 and 2015. As inclusion criterion, studies were considered in English, Spanish and Portuguese, original articles of any type of design, articles with a sample of children and/or adolescents, articles that used the PDSS. Duplicate articles, articles with no relation to the theme, articles with another investigated population, and articles that the parents answered the instrument for their children were excluded. To find the material with these features, the terms “Daytime sleepiness” AND “adolescents” and “Daytime sleepiness” AND “children” were used in the searches. In addition, the descriptor “Pediatric Daytime Sleepiness Scale” was used to filter more specifically.

**Data synthesis:**

Initially, 986 studies related to daytime sleepiness were identified. Considering the inclusion criteria, we analyzed 26 studies composed of 18,458 subjects aged 0 to 37 years. The diurnal sleepiness score ranged from 6.7±0.6 to 25.7±0.6 points. In general, all included studies investigated other sleep variables in addition to daytime sleepiness, such as: sleep duration, sleep quality, sleep hygiene or sleep disorders (narcolepsy and cataplexy), respiratory disorders, neurological and developmental disorders.

**Conclusions:**

There was a moderate use of PDSS to evaluate daytime sleepiness. This instrument allows the monitoring of factors that influence excessive daytime sleepiness in children and adolescents.

## RESUMO

**Objetivo:**

Revisar de modo sistemático a utilização da *Pediatric Daytime Sleepiness Scale* (PDSS) na análise da sonolência diurna em crianças e adolescentes.

**Fontes de dados:**

Foram consultadas as bases de dados eletrônicas PubMed e SciELO, no período delimitado entre 2003 e 2015. Como critério de inclusão, foram considerados estudos em inglês, espanhol e português; artigos originais de qualquer tipo de escopo, com amostra de crianças e/ou adolescentes e que utilizaram a PDSS. Foram excluídos artigos duplicados, sem relação com o tema, com outra população investigada, bem como aqueles em que os pais responderam o instrumento pelos seus filhos. Para encontrar o material dentro desses critérios, foram utilizados nas buscas os seguintes termos: “*Daytime sleepiness*” AND “*adolescents*” e “*Day time sleepiness*” AND “*children*”. Além disso, utilizou-se o descritor “*Pediatric Daytime Sleepiness Scale*” para fazer uma filtragem mais específica.

**Síntese dos dados:**

Inicialmente, identificaram-se 986 pesquisas relacionadas à sonolência diurna. Considerando os critérios de inclusão, foram analisados 26 estudos compostos por 18.458 sujeitos com idades de 0 a 37 anos que responderam à PDSS. A pontuação da escala da sonolência diurna variou de 6,7±0,6 a 25,7±4. Em geral, todos os estudos incluídos investigaram, além da sonolência diurna, outras variáveis do sono, como: duração, qualidade, higiene ou distúrbios do sono (narcolepsia e cataplexia), patologias respiratórias, neurológicas e do desenvolvimento.

**Conclusões:**

Verificou-se moderada utilização da PDSS para avaliar a sonolência diurna. Esse instrumento permite o acompanhamento de fatores que influenciam a sonolência diurna excessiva em crianças e adolescentes.

## INTRODUCTION

Sleep, which is a basic biological process that is essential for the growth and healthy development of children and adolescents,^1^ is considered an important factor for the health of young people. Currently, research in this area has investigated its association both with the proper functioning of cognitive and psychological functions, as well as with metabolic health and obesity. In addition, poor sleep quality has direct repercussions on daytime activities performed by children and adolescents.^2^

Poor sleep quality may lead to excessive sleepiness during the day, with daytime sleepiness being one of the main consequences related to sleep disturbances.^3^ It is characterized by the increased need for napping during the day and has a close relation with declining school performance and with a negative perception of quality of life.^4^

One of the influences in increasing sleep need refers to the biopsychosocial changes that occur in puberty.^5^ Adolescent sleep is characterized by a delay in the timing of sleep due to the innumerable processes of biological, psychic and social changes characteristic of this phase of human development. This delayed sleep onset impairs adaptation to social hours - especially at the end of this period - making it difficult for teenagers to stay awake in situations in which they are required to, such as during school hours, for example.^6^ These situations need to be better investigated since daily routines, school and extracurricular schedules of adolescents, low level of physical activity,^7^ high values of body mass index (BMI), nocturnal awakening^4^ and respiratory problems^8^ are predictors of pathologies associated with daytime sleepiness.

The most accurate method for assessing daytime sleepiness is the Multiple Sleep Latency Test (MSLT). Considered a gold standard, it is performed in laboratory settings and aims to evaluate how quickly a patient falls asleep in a soporific situation, as well to verify abnormal transitions between wakefulness and REM sleep.^8^ However, it is a high-cost resource with difficult implementation in field research.^9^ Thus, the need to quantify daytime sleepiness subjectively through self-reporting measures is understandable. The most widely used instrument for this purpose is the Epworth Sleepiness Scale.^10^ However, in its original version, such scale is suitable only for the adult population. Moreover, even without validation for children and adolescents, with the exclusion of questions that consider situations that do not represent the daily life of these populations,^11^ ^,^ ^12^ the Epworth Scale has already been used in a modified way.^13^ In this context, the Pediatric Daytime Sleepiness Scale (PDSS) has been recently validated^14^ and translated into Portuguese by Felden et al.^15^ to be used in the investigation of daytime sleepiness in Brazilian children and adolescents. It is a self-assessment instrument that describes some daily life situations related to sleep habits, waking time and sleep problems.^4^

Therefore, this work aimed to systematically review the use of PDSS in the analysis of daytime sleepiness in children and adolescents. Thus, it also aimed to make progress in research, seeking to identify general scores observed, associated factors and main results. By doing so, we were able to group data to support the viability of application of the PDSS instrument in research.

## SOURCES OF DATA

**Figure 1:**
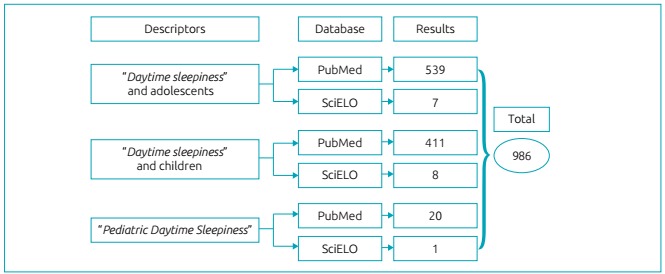
Database Search Strategy.

We selected PubMed and SciELO electronic databases to perform the searches. The reasons for this choice included good criteria for periodic evaluation and an impact factor measurement based on international standards of scientific communication and a variety of articles in the area of health. In order to find studies that used PDSS specifically for children and adolescents, we initially typed the keywords “daytime sleepiness”, “adolescents” and “children” combined with the Boolean operator “AND”. In addition, in order to filter the search, we used the descriptor “Pediatric Daytime Sleepiness Scale” according to Figure 1.

To make our search more specific, we delimited the date (2003-2015) and only selected works involving human subjects. Inclusion criteria for the present review included:

1. Texts in English, Spanish or Portuguese;
2. Original articles, all designs eligible;
3. A sample consisting of children and adolescents;
4. Daytime sleepiness evaluated with PDSS.

An article selection process to compose the review occurred in four stages:

1. Database search;
2. Title review;
3. Title skimming;
4. Reading articles in full.

Initially, we excluded duplicate studies and those that clearly were not related to the subject of the present review - for example, those evaluating a different population (teachers, workers, and drivers). We also excluded studies in which parents answered the questionnaire for their children. All the steps were carried out by two evaluators, who discussed the suitability of the articles according to the established criteria. In cases of disagreement, a third evaluator was consulted.

To assess article quality, we used the rating scale proposed by Downs and Black,^16^ consisting of 27 questions that estimate communication, external validity, internal validity (bias and confusion) and statistical power. For the present study, all of the questions for the intervention articles were used; questions 8, 13-15, 17, 19, 21, 22-24 and 26 were excluded for the evaluation of cross-sectional articles; questions 8, 13-15, 17, 19, 23 and 24 were excluded for control case studies; and questions 8, 13-15, 19, 21-24 were excluded for longitudinal studies. According to the quality evaluation proposal, the questions were scored 0 or 1, except question 5, which ranged from 0-2 points. In addition, question 27, which analyzes the statistical power, ranged from 0-5. Thus, according to the adaptation performed for each article of different design, an intervention study could obtain a maximum score of 32 points, cross-sectional studies 21 points, control cases 24 points and longitudinal studies 23 points. 

We followed the recommendations of Costa et al.^17^ to obtain a detailed analysis of the methodology of this systematic review, in addition to a better methodological description. We observed all the criteria for the types of searches and contents contained in a systematized review.

## DATA SUMMARY

**Figure 2:**
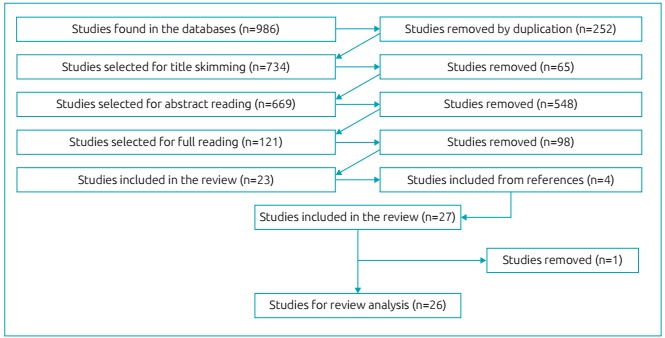
Flowchart of the selection process of articles that composed the review.

We found 986 studies related to daytime sleepiness in children and adolescents, as described in Figure 1. Of these, 252 articles were excluded because they were duplicated, leaving 734 studies for title skimming. After the titles were read, 65 were excluded because they did not fit the inclusion criteria. Thus, 669 studies were selected for abstract reading and, of these, 548 abstracts were excluded because they did not present an evaluation of daytime sleepiness in their methods and/or results. Therefore, 121 articles were left for reading in full. Of these, 98 were excluded because they did not use the instrument analyzed in the present review. Thus, 23 articles met the inclusion criteria and other 4 articles were selected from the references. Of these 27 articles, 1 was excluded because the result was not in accordance with the objective of the present review. Therefore, 26 studies^18^ ^,^ ^19^ ^,^ ^20^ ^,^ ^21^ ^,^ ^22^ ^,^ ^23^ ^,^ ^24^ ^,^ ^25^ ^,^ ^26^ ^,^ ^27^ ^,^ ^28^ ^,^ ^29^ ^,^ ^30^ ^,^ ^31^ ^,^ ^32^ ^,^ ^33^ ^,^ ^34^ ^,^ ^35^ ^,^ ^36^ ^,^ ^37^ ^,^ ^38^ ^,^ ^39^ ^,^ ^40^ ^,^ ^41^ ^,^ ^42^ ^,^ ^43^ were included and completely analyzed in our study. Figure 2 shows the selection process of the articles.

Most articles (n = 14) were published in recent years (2012-2015). Sample size varied from 22-7,556 children and adolescents of both sexes. All the studies included in the review were carried out outside Brazil, since Brazilian surveys did not meet the criteria established for the present review.

The quality evaluation of the selected studies was described in Table 1. The median score, according to the Downs and Black criteria,^16^ was 12.2 (with a minimum of 9 and a maximum of 15 points). The mean score of the analyzed studies was 11 ± 2 points. Regarding the methodological evaluation, the questions related to the communication domain (clarity in the description of objectives, confounding variables, probability values) were those that best met the criteria proposed for quality analysis, presenting higher score averages. However, the questions that indicated external validity were those that presented greater methodological limitations, with low inclusion rates. It should be noted that of the 26 articles, only 4 presented statistical power; 6, confounding factors; and 8, adjustments of confounding analysis.

**Table 1:**
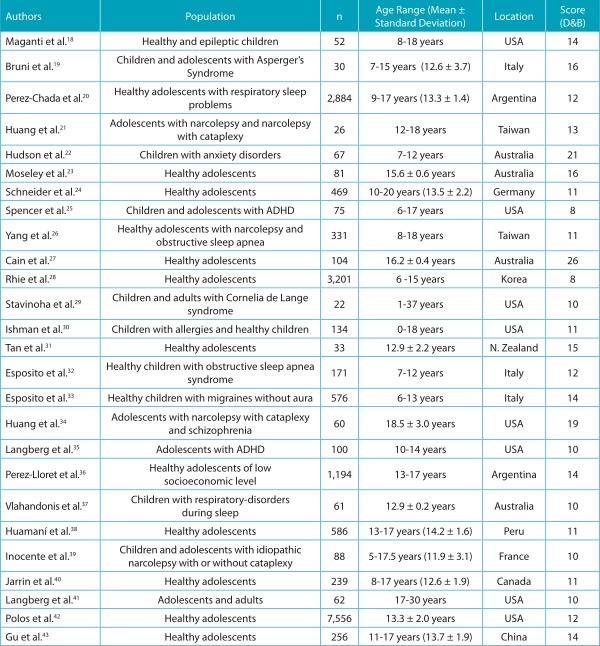
Characteristics of the articles included in the review that analyzed the Pediatric Daytime Sleepiness Scale.

Score (D&B) = Downs and Black criteria. *ADHD: Attention deficit hyperactivity disorder.

Most of the studies used PDSS as an instrument to assess sleepiness related to sleep disorders (respiratory, neurological, and developmental disorders) and to assess sleepiness side effect monitoring in pharmacological treatments. In some studies, the authors applied the scale to healthy children and adolescents. Scale scores ranged from 6.7 ± 0.6-25.7 ± 4.6, showing in some studies (n = 6) a tendency for excessive daytime sleepiness. Among the studies, 15 performed experimental research, evaluating groups (experimental and control) to identify the differences between the diseases and daytime sleepiness in participants.

Table 2 shows designs, investigated factors and means, and standard deviation of the sum of the PDSS questions of all the groups surveyed. Noting that the instrument does not have cut-off points for classification, the studies considered the mean of the scale score.

**Table 2:**
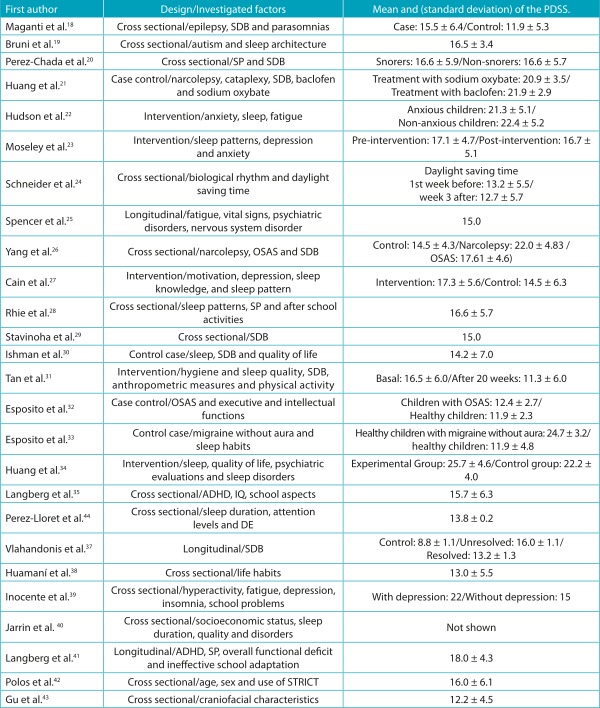
Design, investigated factors, mean and standard deviation of the Pediatric Daytime Sleepiness Scale of the studies included in the present review.

*PDSS: Pediatric Daytime Sleepiness Scale; β: Coefficient beta; ADHD: attention deficit hyperactivity disorder; IQ: Intelligence quotient; OSAS: obstructive sleep apnea syndrome; N-C: Narcolepsy with cataplexy; STRICT: Sleep time-related information and communication technology; SP: school performance; SDB: sleep-disordered breathing.

All studies included investigated, in addition to daytime sleepiness, other sleep variables such as sleep duration, sleep quality, sleep hygiene or sleep disorders. In contrast, sleep disorders (narcolepsy and cataplexy) were the most prominent in comparison to respiratory diseases (breathing problems, obstructive sleep apnea syndrome) and neurological and developmental disorders (attention deficit hyperactivity disorder). The results of each study are shown in Table 3.

**Table 3:**
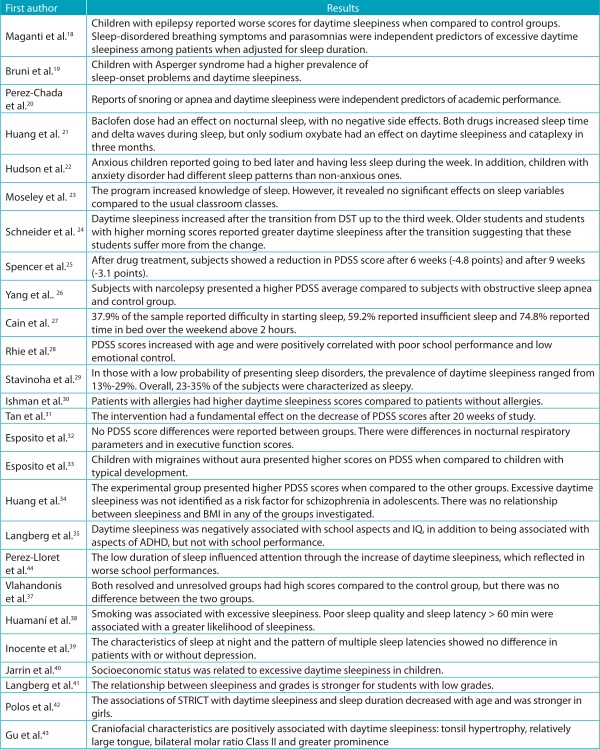
Main results found in the studies included in the review.

*PDSS: Pediatric Daytime Sleepiness Scale; ADHD: Attention Deficit Hyperactivity Disorder; STRICT: Sleep time-related information and communication technology; IQ: Intelligence Quotient.

## DISCUSSION

Experiencing somnolence events during the day is natural. However, the worsening of this behavior can lead to unsatisfactory levels of sleep and related disorders, which can be characterized as excessive daytime sleepiness. This disorder is one of the most frequent complaints related to sleep, affecting between 10-25% of the population.^44^ ^,^ ^45^ According to the literature, both the difficulty in staying awake and alert during the day,^14^ ^,^ ^45^ ^,^ ^46^ and the increased subjective perception of sleep need^4^ are characterized as excessive daytime sleepiness. This disorder results in involuntary naps and lapses during sleep. Excessive daytime sleepiness is more likely to occur in monotonous situations of daily living or in situations of risk. It is linked to negative social, professional, and family effects, to a decrease in work and school performance, to low learning outcomes and to reductions in quality of life.^4^ ^,^ ^45^ ^,^ ^47^

The main factors that contribute to excessive daytime sleepiness are: low duration and poor sleep quality, irregular sleep and wake patterns, medical or neurological conditions associated with direct impact on sleep (depression, anxiety, epilepsy, among others), the use of psychoactive substances and the presence of primary hypersomnia.^48^ In addition to these, other factors that could contribute to this variable, such as sedentary behavior and physical fitness, require further study. Among the pathological causes and primary sleep disorders are: obstructive sleep apnea syndrome (OSAS) - with a prevalence of 2% in women and 4% in men; central sleep apnea syndrome; narcolepsy - 0.02-0.18% of the population; idiopathic hypersomnia - 10% of patients suspected of narcolepsy; inadequate sleep hygiene; restless legs syndrome (REM sleep behavioral disorder); periodic limb movement disorder (PLMD); and circadian rhythm disorders (delayed and advanced sleep phase disorders).^49^

There are several ways to assess daytime sleepiness. The main method of objective evaluation of excessive daytime sleepiness is MSLT, which is used for the diagnosis of narcolepsy and idiopathic hypersomnia. However, because it is a high-cost test, subjective evaluations are usually performed through questionnaires and sleep diaries. The advantages of these subjective evaluations are the low cost and ease of application. Although these types of standardized assessments promote uniformity in the subject’s approach, their use is limited to subjects with intellectual disabilities.^50^

Thus, the studies selected to compose this review used the subjective self-report questionnaire, which includes an adequate evaluation for children and adolescents, the PDSS.^14^ The questionnaire has 8 four-point Likert scale items with a total score varying from 0-32. Of the studies analyzed, 6 showed a prevalence of negative sleep patterns such as excessive daytime sleepiness, with results close to 32 points (20.9 ± 3.5-25.7 ± 4.6 points).^21^ ^,^ ^22^ ^,^ ^26^ ^,^ ^33^ ^,^ ^34^ The rest of the studies presented mean values of 6.7 ± 0.6-18.0 ± 4.3 points.^17^ ^,^ ^20^ ^,^ ^21^ ^,^ ^22^ ^,^ ^25^ ^,^ ^26^ ^,^ ^27^ ^,^ ^29^ ^,^ ^30^ ^,^ ^31^ ^,^ ^32^ ^,^ ^33^ ^,^ ^34^ ^,^ ^37^ ^,^ ^38^ ^,^ ^42^ ^,^ ^43^ Because it is a quantitative scale with a defined value and because it does not contain a cut-off point as a predictor of excessive daytime sleepiness, it is inferred that the score near the upper limit of the scale reflects evidence of excessive daytime sleepiness.

Pereira et al.^51^ reported that low sleep duration is one of the main predictors of excessive daytime sleepiness when they observed that Brazilian adolescents needed to sleep at least 8.3 hours on school days as a protection against excessive daytime sleepiness. Similarly, one of the analyzed studies - conducted by Huang, Wang and Guilleminault with 1,939 adolescents 12-18 years of age from northern Taiwan,^21^ describing sleep problems - found that the mean duration of sleep (7.3 ± 1.2 hours) presented a negative correlation with the PDSS total score in the age groups of 14-15 years and 16-18 years. In addition, other studies on the prevalence of daytime sleepiness identified the presence of this symptom in 25% of university students aged 17-24 years,^52^ 35.7% of adolescents up to 21 years^51^ and 40% of adolescents aged 12-19 years.^53^ However, it is worth mentioning that, in the studies analyzed in the present review, the majority had as an object of study, the use of PDSS in children and adolescents with some associated disease. As for example, a study by Stavinoha et al.^29^ in patients with Cornelia de Lange syndrome pointed out the presence of this symptom in 23% of individuals under 15 years of age and in 36% of those above that age.

Regarding research design, most articles were cross sectional studies (n = 14). A few had intervention (n = 5), control (n = 4) or longitudinal (n = 3) designs. In addition, only 2 studies applied follow-up analyzes to their individuals.^23^ ^,^ ^37^ The systematic analysis and behavior monitoring of daytime sleepiness over time is considered of paramount importance to establish cause and effect relationships. The scarcity of studies with a longitudinal design in the present analysis reveals certain fragility in their conclusions.

In addition to PDSS, other instruments also propose to evaluate excessive daytime sleepiness. In the articles that contribute to this review, authors used the Sleep Disturbance Scale for Children^54^ to evaluate sleep habits and disorders, and the Epworth Sleepiness Scale to verify daytime sleepiness in everyday situations in adults^10^ and children.^13^

A relevant point is the relation between sleepy young people and school performance. This relationship was verified in a study by Perez-Chada et al.^20^ that showed a significant association between daytime sleepiness and school failure. In addition, other studies have also identified this association^20^ ^,^ ^28^ ^,^ ^35^ ^,^ ^41^ ^,^ ^44^

We observed a special concern of researchers in carrying out analyzes to identify key factors that can cause excessive daytime sleepiness in children and adolescents with or without disorders. The studies that used PDSS as an evaluation instrument investigated relationships of daytime sleepiness with various disorders and associated factors, such as school performance and life habits. Thus, this review opens the field for a deeper evaluation of the association between physical activity practice and sedentary behaviors with excessive daytime sleepiness in children and adolescents.

## CONCLUSION

We observed that PDSS is a widely used instrument for assessing daytime sleepiness and that, through its questions, it is possible to identify factors related to excessive daytime sleepiness in children and adolescents. Scale scores ranged from 6.7 ± 0.6-25.7 ± 4.6. The main factors associated with daytime sleepiness in the investigated literature were the short sleep duration, occurrence of sleep disorders and, therefore, poor school performance. Despite limitations in the literature, such as the scarcity of studies involving Brazilian children and adolescents, and the lack of proposals for possible cut-off points to discriminate excessive daytime sleepiness, PDSS has proved to be feasible for research with children and adolescents because of its easy application and good understanding among adolescents.
